# Supplementary material for: Predicting mechanical properties of CFRP composites using data-driven models with comparative analysis
Source: PLoS One. 2025 Apr 7;20(4):e0319787. doi: 10.1371/journal.pone.0319787 (PMC11975104; doi:10.1371/journal.pone.0319787)
Supplement: S1 File — This file contains supplementary results on multilinear regression analysis, including statistical assumption checks, collinearity analysis, and a correlation matrix. S1 Fig. Validity of the regression’s assumptions. S2 Fig. Durbin-Watson test. S3 Fig. Tensile stress-strain curves for 5 types of CFRP composites. S4 Fig. Scanning electron microscopy (SEM) of CFRP samples with epoxy foamed interlayer. S1 Table. Summary of the p-values corresponding to each dependent variable where x1: carbon nanotube volume fraction, x2: interlayer volume fraction, x3: glass transition temperature and x4: manufacturing pressure. S2 Table. Correlation matrix of independent variables where x1: carbon nanotube volume fraction, x2: interlayer volume fraction, x3: glass transition temperature, and x4: manufacturing pressure. S3 Table. Tensile modulus for five types of CFRP composites. (DOCX) [file pone.0319787.s001.docx]

# **Supplementary Material**

Multilinear Regression:

Multi-linear regression models were generated using the R software [1] for the flexural strength and modulus as functions of carbon nanotube volume fraction, interlayer volume fraction, glass transition temperature, and manufacturing pressure. For the flexural strength model an R^2^ of 96.55% was achieved with LOOCV, all assumptions checked, and collinearity not detected. The statistical significance of each independent variable was evaluated using p-values, as shown in S1 Table.

S1 Table : Summary of the p-values corresponding to each dependent variable where x_1_: carbon nanotube volume fraction, x_2_: interlayer volume fraction, x_3_: glass transition temperature and x_4_: manufacturing pressure.

| Dependent variables | P-Values | | | |
| --- | --- | --- | --- | --- |
|  | $x_{1}$ | $x_{2}$ | $x_{3}$ | $x_{4}$ |
| Flexural Strength | 0.00251 | 2.57$\times{10}^{-11}$ | < 2$\times{10}^{-16}$ | < 2$\times{10}^{-16}$ |
| Flexural Modulus | 0.451 | 8.85$\times{10}^{-14}$ | 5.55$\times{10}^{-14}$ | 4.01$\times{10}^{-14}$ |

Assumptions for multilinear regression models:

The first assumption to check was the normality of errors, which allows for determining if the dataset can be modeled with a normal distribution. For this purpose, a standardized residual plot and its histogram were reviewed. The standardized residual plot and histogram, shown in S1 Fig. , demonstrate a normal distribution that denotes the normality of the dataset.

The second assumption was homoscedasticity, also known as homogeneity of variance. Data is homoscedastic if the residuals plot is the same width for all values of the predicted outputs, which can be seen if there is a lack of discernible shape or direction to the plot of the standardized residuals against the standardized predictions plot. The output residual plot does not show any specific shape, which denotes normality (S1 Fig. ).


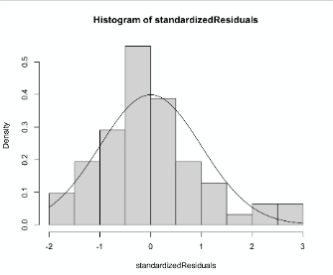

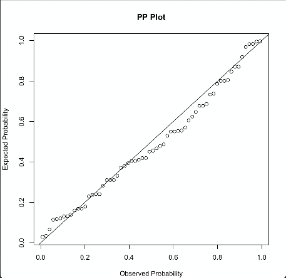


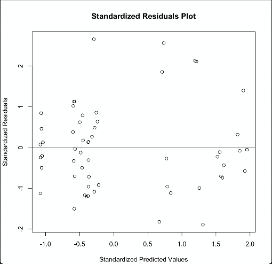


S1 Fig. : Validity of the regression’s assumptions.

The third assumption is the independence of error, which means that there is no relationship or autocorrelation between the residuals and flexural strength values. The Durbin-Watson test is used to check for the independence of error. S2 Fig. shows the potential outcomes of the Durbin-Watson test. The testing concluded a DW value of 2.18, which falls between the “no autocorrelation” ranges of 1.5 and 2.5 and denotes normality. Since all three assumption checks yielded positive results that denote the normality of the dataset, the linear regression can represent the model.


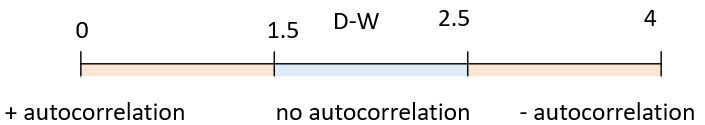


S2 Fig. : Durbin-Watson test.

Collinearity:

Multicollinearity means that one of the independent variables in the model can be calculated using the other independent values. It is important to ensure no collinearity as it can affect the model's accuracy. Values of ± 0.7 in the correlation matrix may indicate collinearity. A matrix for the model is developed using R, as shown in S2 Table , demonstrating that all correlations are less than 0.7, thus suggesting no correlation between independent variables.

S2 Table : Correlation matrix of independent variables where x_1_: carbon nanotube volume fraction, x_2_: interlayer volume fraction, x_3_: glass transition temperature, and x_4_: manufacturing pressure.

|  | $x_{1}$ | $x_{2}$ | $x_{3}$ | $x_{4}$ |
| --- | --- | --- | --- | --- |
| $x_{1}$ | 1.000 | 0.570 | -0.361 | -0.262 |
| $x_{2}$ | 0.570 | 1.000 | -0.621 | -0.458 |
| $x_{3}$ | -0.361 | -0.621 | 1.000 | 0.693 |
| $x_{4}$ | -0.262 | -0.458 | 0.693 | 1.000 |

Another way to determine collinearity is using the variance inflation factor (VIF). The VIF measures how much an independent variable's behavior (variance) is influenced or inflated by its interaction/correlation with the other independent variables. The VIF was used to verify the conclusion from the correlation matrix. All VIF values for the independent variables were below the threshold value of 10, which further proves that there is no collinearity in the independent variables.

# **References**

[1] "RStudio | Open source & professional software for data science teams", *Rstudio.com*, 2022. [Online]. Available: https://www.rstudio.com/. [Accessed: 12- Jan- 2022].
