# Supplementary material for: Predicting mechanical properties of CFRP composites using data-driven models with comparative analysis
Source: PLoS One. 2025 Apr 7;20(4):e0319787. doi: 10.1371/journal.pone.0319787 (PMC11975104; doi:10.1371/journal.pone.0319787)
Supplement: Attachment — Submitted filename: Response to Reviewers.docx [file pone.0319787.s003.docx]

We thank the editor for their feedback. The following items are being submitted with the revised manuscript:

• A rebuttal letter that responds to each point raised by the academic editor and reviewer(s).

• A marked-up copy of the manuscript that highlights changes made to the original version. You should upload this as a separate file labeled 'Revised Manuscript with Track Changes'.

• An unmarked version of your revised paper without tracked changes. You should upload this as a separate file labeled 'Manuscript'.

1. Please ensure that your manuscript meets PLOS ONE's style requirements, including those for file naming. The PLOS ONE style templates can be found at https://journals.plos.org/plosone/s/file?id=wjVg/PLOSOne_formatting_sample_main_body.pdf and <https://journals.plos.org/plosone/s/file?id=ba62/PLOSOne_formatting_sample_title_authors_affiliations.pdf>

- The references style was updated to the Vancouver style as recommended by the journal.

- Line numbering has been incorporated.

- Title and author affiliations formatted according to the style.

- “Figure” replaced with “Fig”

2. Please note that PLOS ONE has specific guidelines on code sharing for submissions in which author-generated code underpins the findings in the manuscript. In these cases, we expect all author-generated code to be made available without restrictions upon publication of the work. Please review our guidelines at https://journals.plos.org/plosone/s/materials-and-software-sharing#loc-sharing-code and ensure that your code is shared in a way that follows best practice and facilitates reproducibility and reuse.

Our data has been published and we are happy to provide the code upon request.

The following section has been added:

**Data availability**

The data used in this manuscript is publicly available at Mendeley Data [69] and python codes will be made available upon request.

3. We note you have included a table to which you do not refer in the text of your manuscript. Please ensure that you refer to Table 1 in your text; if accepted, production will need this reference to link the reader to the Table.

This has been fixed in the revised manuscript as:

Table 1 lists the nomenclature for all the abbreviations used in this manuscript.

4. Please include captions for your Supporting Information files at the end of your manuscript, and update any in-text citations to match accordingly. Please see our Supporting Information guidelines for more information: <http://journals.plos.org/plosone/s/supporting-information>.

- Supplementary materials document has been renamed to “Supporting information”.

- Captions were included for the supporting information in the revised version of the manuscript.

Responses to reviewers:

We thank the reviewers for the excellent feedback which will improve the quality of our paper. We here try our best to respond to the comments of the reviewer one-by-one. We also made highlighted modifications inside the manuscript, to directly address the comments of the reviewers.

Reviewer #1: Following points should be considered

• Please mention the size of the dataset used in training the models in the Abstract

The sample size has now been included in the abstract of the revised version.

• From Section 1. Introduction: "In polymer reinforced composites, the polymer matrix controls thermal and chemical properties, while the reinforcing material determines the mechanical properties" → Please provide some examples of these "thermal and chemical properties" and "mechanical properties"

Examples have been added in the revised version as recommended by the reviewer:

In general, for polymer-reinforced composites, the polymer matrix controls thermal and chemical properties such as glass transition temperature, corrosion resistance, and chemical stability [2,3], while the reinforcing material controls mechanical properties such as tensile strength and impact resistance [4–7].

• From Section 1. Introduction: "replacing metal-based materials in low-temperature applications due to these properties" → Please explain what these "low-temperature applications" are.

Explanations have been added in the revised version as suggested by the reviewer:

“Due to these properties, carbon fiber-reinforced polymer (CFRP) composites have been crucial in replacing metal-based materials in low-temperature applications such as thermal insulators [9], electronic packaging [10], biomedical implants [11], and cryogenic systems [12].”

• From Introduction: "More recently, machine learning (ML) has become widely spread in predicting the mechanical properties of composite materials, significantly saving experimental and computational costs[11,12]" → At this point the authors should provide a much more comprehensive list of relevant publications since ML methods have been extensively used in the predictive modeling of composite materials. I recommend adding the following recent publications:

https://doi.org/10.1038/s41598-021-85963-3

<https://doi.org/10.3390/ma16134578>

<https://doi.org/10.1002/pc.27969>

<https://doi.org/10.3390/biomimetics9090544>

<https://doi.org/10.1016/j.compositesb.2023.111132>

We agree with the reviewer. The suggested recent publications have been added to the revised version of the manuscript with additional text:

“More recently, machine learning (ML) has become widely spread in predicting the mechanical properties of composite materials, significantly saving experimental and computational costs [6,7,20–24]. The application of ML also provided efficient solutions for the traditional difficulty associated with carrying out computational mechanics simulations involving multiscale and nonlinear behavior common in composites [6,7,22–24].”

• From Introduction: "Pathan et al. [17] found that the time required to predict the mechanical properties using a machine learning model is 3600 times faster than using a finite element model." → This statement should be justified and further details should be provided about the measurement procedure. Although predicting the behavior of a model using trained models can be a fast process, the ML process also includes the data collection, preprocessing and model validation stages. Looking at the issue from this perspective, I don't think ML process is 3600 times faster than finite element analysis. Although finite element model building can be a time consuming process, once a model has been validated, running structural analysis simulations is a very fast process as well. Therefore, I recommend including more detailed information about in what way the ML process is faster than finite element analysis.

The number reported “i.e. 3600” here refers only to the prediction stage after building and validating the FE and ML models. We have added more explanation to the revised version of the manuscript as recommended by the reviewer.

“Pathan et al. [29] found that the time required to predict five mechanical properties using a machine learning model was about 0.5 seconds, which was 3600 times faster than using a finite element model that took about 30 minutes to make the same predictions. This excludes the time required to build and validate the FE model or the time required for data collection, preprocessing, training and validation of the ML model.”

• I recommend, if available, including the actual pictures of the experimental setup in addition to drawing in Figure 1 and Figure 3. Also, Figure 1 appears 2 times. Please remove one of them

Pictures have been added as recommended. Please note that we are submitting the revised version of the article without the figures and attaching the figures separately.

• In addition to hardware used in training the models, please also mention the software platform used such as Scikit learn, Tensorflow etc.

Software packages have been added in the revised version of the manuscript:

“Pandas, scipy, and scikit-learn python packages were used to process the data and build the ML models.”

• In the Discussion section the authors are proposing predictive formulas which should be done earlier in the text. I suggest moving that part into Results section or into a separate section before Discussion.

We move the predictive formulas with the corresponding text to end of the Results section as suggested by the reviewer.

Reviewer #2: Alsheghri et al. applied ML models to predict polymer matrix-carbon reinforced composites (PMCRC). The input variables were manufacturing method and volume fiber content, whereas the output variables were 3-point flexural strength and modulus and the mode II energy release rate. Prediction metrics was improved after including manufacturing type. However, experimental details are missing; the literature review left out some recent developments / currently accepted methods and needs more critique of the current state. Some other issues need to be resolved before it can be considered for publication.

- Experimental details are missing: load cell, extensometer/deflection gauge model, mouth opening gauge/crack length measurement device, acquisition and processing software

The following experimental details have been added to the revised version of the manuscript.

“The specimens were tested in a screw-operated Instron machine (model 4505). Mechanical tests were performed from no loading state to failure. The following parameters were used for the flexural and mode II experiments: Data Acquisition Rate 10 point/sec, Crosshead Speed 5.0 mm/min, Temperature 23 °C, and Humidity 50%. A 10 kN load cell was used for the flexural & mode II tests (model 2518-603). The parameters for the tensile experiments were: Data Acquisition Rate 10 point/sec, Crosshead Speed 1.0 mm/min, Temperature 23 °C, and Humidity 50%. A 100 kN load cell was used for the tensile tests (model 2518-611) and a strain gauge extension-meter (model 2620-827). The Bluehill Materials Testing Software was used for acquisition and Origin software was used for processing.”

- Fig 1, Fig 2 are repeated. It probably happened when you duplicate the marker in MS-Word.

This issue has been fixed in the revised manuscript in which we just include the captions of the Figures. The Figures are now submitted separately to avoid this issue or other problematic issues.

- Fig 8b ... Feature premutation ....

Figure 8 in text was renamed to “Figure 8b”.

- Are the bending samples manufactured/tested according to a standard? Please state that explicitly.

Yes, we now mentioned that:

“The samples used to measure the flexural strength were manufactured and according to the standard test method for the composite materials ASTM D7264 for a three-point load test on a simply supported beam that uses central loading”

- Major: The literature revision seems to focus on speed prediction rather than prediction accuracy. Furthermore, some accepted models, such as the Voight rule or micromechanics, are used for the same tasks that are not mentioned.

The literature review was expanded in the revised version of the manuscript as recommended. The following text was added:

- “Numerical cross-scale simulations, including finite element analysis (FEA) [18], offer a much quicker and less costly method compared to synthesizing, developing, and testing materials in a laboratory. Previous work used homogenization theory [19] and micromechanics [20] to predict the mechanical properties of composite materials. While the homogenization theory approximates the behavior of heterogeneous materials by treating them as if they were homogeneous, micromechanics models such as the rule of mixtures use the principles of mechanics to describe how the fibers and matrix interact to affect the overall properties of the composite. These models are rooted in physical understanding and involve detailed consideration of the composite's microstructure, including the distribution of fibers, matrix properties, fiber-matrix interactions, and load transfer mechanisms [21]. However, their accuracy can be limited by simplifying assumptions such as homogeneity of material phases [20] or the uniformity of strains (e.g. Voigt model) [22]. While they are generally based on fundamental physics, micromechanics models can struggle with highly complex or heterogeneous materials without significant adjustments or additional assumptions [23].”
- “While ML models, especially deep learning, often encounter challenges to explain the underlying physical mechanisms, micromechanics models provide clear insights into the physical processes at the microstructural level, allowing the understanding how different factors (e.g., fiber alignment, matrix properties) affect the overall mechanical properties of the composite [47]”.
- The authors left recent advances in mechanical properties prediction using ML (Prada 2024), who used some of the models tested here for similar tasks. They use non-linear algorithms, which proved to be a better solution when predicting strength and elasticity (Prada 2024). Moreover, Random Forest and NN, are known for overfitting data. .***Prada-Parra, D. Supervised Machine Learning Models for Mechanical Properties Prediction in Additively Manufactured Composites. Appl. Sci. 2024, 14, 7009. <https://doi.org/10.3390/app14167009>
- We thank the reviewer for referring to this recent publication. Kindly note that we missed this article because it was published around the time our manuscript was submitted to the journal. We now refer to this article in the revised version of the manuscript.
- The machine learning models considered in our study were carefully selected to cater for a small amount of training data. Unlike the work of (Prada, 2024) which collected data from the literature, our study depended on experimental data provided by the industrial partner, which limited the amount of number of samples in our dataset. The selected ML models provide a high degree of model generalization, which is highly important for limited data. Given our relatively small dataset, we did not use NN to avoid overfitting.
- In addition, given the smaller number of features (i.e. 5 independent features) and the different nature of the considered problem, it was important to compare linear versus nonlinear ML models. Our results showed that for certain properties such as flexural strength and flexural modulus, both linear (ridge regression) and nonlinear (random forest) models provided comparable results (see Table 3). We selected the random forest for the nonlinear model due to its Ensemble characteristic (combining predictions from 100 decision trees). Ensembles generally reduce the chance of overfitting and generate more robust predictions than single models. The RF model was also selected by (Prada, 2024) and demonstrated the best results in terms of RMSE. We elaborated on this point in the revised version of the manuscript as follows:

“In addition, our results showed that for certain properties such as flexural strength and flexural modulus, both linear (ridge regression) and nonlinear (RF) models provided comparable results (see Table 3). Table 3 also shows that for mode II energy release rate, the RF model yielded the highest R2 value. Being an Ensemble model, the RF generally reduces the chance of overfitting. The RF model was also reported by [62] for demonstrating best results in predicting the tensile strength and elastic modulus for continuous fiber-reinforced polymer matrix composites.”

- The authors performed tensile tests but omitted the tensile elastic modulus from calculation and prediction. Such comparison between flexural and tensile elastic modulus is always helpful, with the significance of each one depending on the PMCRC´s specific application.
- The energy release rate is crucial for fail-safe designs. It makes sense that the manufacturing method impacts it, as the matrix-fiber bond might be enhanced at higher pressures. The predictions shown in Figs 7 and 8 seem very tight below 1000 kJ/m^2 but scattered above that value. Without experimental details on how it was calculated, one cannot venture to guess the origin of the high dispersion at higher rates. I urge the authors to discuss such behavior.

Both of the above comments have to do with the interlayered specimens. Specimens that were reinforced with interlayer between the carbon fiber successive layers. The provided data was from multilayer CFRPs as following:

1. CFRP with not interlayer (Type 1) - control
2. CFRP with epoxy foam interlayer (Type 2) - EPF
3. CFRP with epoxy foam and CNTs in the interlayer (Type 3) – EPF_CNT
4. CFRP with electrospun nanofibers in the interlayer, (Type 4) and – ELSP
5. CFRP with electrospun nanofibers and CNTs in the interlayer (Type 5) – ELSP_CNT
(i) As it can be seen from the graphs presented below, during the tensile testing the carbon fiber was dominant, so there was no significant change in the tensile modulus and this is why the tensile modulus was omitted from our manuscript. Nevertheless, the tensile strength was included in our analysis, because in the case of electrospun interlayer an increase of tensile strength was observed (about 3%), which is attributed to the extra load-transfer mechanisms that nanofibers introduce to the CFRP composite.

(Type 1)	(Type 2)

(Type 3)	(Type 4)

(Type 5)

| Type | Tensile Modulus [GPa] |
| --- | --- |
| Control CFRP | 9.83±2.6 |
| CFRP+EP.FOAM | 10.1±1.2 |
| CFRP+EP.FOAM+CNT | 8.83±0.8 |
| CFRP+ELSP | 8.43±0.6 |
| CFRP+ELSP+CNT | 9.27±0.3 |

The figures and table were added in the supporting information.

(ii) In the case of energy release rate, the increased scattering is owed to the epoxy foamed interlayer, especially with CNTs (Type 2 and 3 in the above list), which were observed to have such a large scattering. This phenomenon is explained by the fact that the foaming interlayer is “non-homogeneous” both in terms of voids distribution within the foaming interlayer and in terms of voids size. During Fracture Toughness Mode II experiments, the crack starts propagating within the interlayer such that:
     1. In certain cases, the crack can reach to a CNT reinforced “small” void, which can be a crack inhibitor requiring more energy for the crack to propagate, resulting in high energy release rates.
     2. In other cases, the crack can propagate between the voids without reaching a void or reach to “large” voids which, regardless of their reinforcement with CNTs or not, facilitates the crack propagation, resulting in lower energy release rates.

Please refer to the pictures below of the epoxy foamed interlayers under SEM microscopy. These pictures were added in the supporting information.

The following text was included in the revised version of the manuscript:

“Our experimental work also revealed no significant change in the tensile modulus during the tensile testing because the fibers were dominant in the tensile load transfer. Therefore, the tensile modulus results were not included. The average tensile modulus of the samples was approximately 9.29 GPa. Nevertheless, the tensile strength was included in our analysis, because in the case of electrospun interlayer an increase of tensile strength was observed (about 3%), which is attributed to the extra load-transfer mechanisms that nanofibers introduce to the CFRP composite (see S3 Figure and S3 Table in the supporting information). The epoxy foamed interlayer, especially with CNTs was observed to cause an increase in the values of energy release rate with large scattering. This phenomenon is explained by the fact that foaming interlayer is “non-homogeneous” both in terms of voids distribution within the foaming interlayer and in terms of voids size (see S4 Figure in the supporting information). During fracture toughness mode II experiments, the crack starts propagating within the interlayer in different manners: (1) In certain cases, the crack can reach to a CNT reinforced “small” void which can be a crack inhibitor requiring more energy for the crack to propagate and resulting in high energy release rates. (2) In other cases, the crack can propagate between the voids without reaching a void or reach to “large” voids, which facilitates the crack propagation resulting in lower energy release rates.”
